# Boiling point: the health risks of heat protection restrictions for agriculture and construction workers

**DOI:** 10.3389/fpubh.2024.1484975

**Published:** 2024-11-20

**Authors:** Chase DeLong, Catherine Marudo

**Affiliations:** Department of Public Health Sciences, Miller School of Medicine, University of Miami, Miami, FL, United States

**Keywords:** heat-related illness, occupational health, urban heat island, legislation, climate change, construction, agriculture, OSHA

## Introduction

The afternoon commute was uniquely uncomfortable. I parked my car on the third floor of the Florida parking garage and immediately felt hot air engulf my body, sweat slowly soaking my freshly laundered scrubs. Relief came as I entered the frigid emergency department, ready to begin my afternoon shift. My first patient was LJ, a young man coming in complaining of worsening headaches, debilitating fatigue, and uncomfortable muscle cramps. As a medical student, I astutely performed a thorough history, presented his story to my attending, and suggested a basic metabolic panel to see if that was a source of his headache and fatigue. After 30 min, his lab results showed extremely low Na+ and K+ and a concerningly elevated BUN/Creatine ratio, indicating that LJ's kidneys were distressed. In a panic, I hastily walk back to his bay, remembering that I forgot to ask LJ about an essential part of his history: what he did for a living.

Climate change is often silent and underappreciated, but it is an especially salient threat to the global workforce. In the case of LJ, he had been working as a landscaper for a private company, and the summer of 2023 marked the hottest year on record at the time, with 2024 continuing to break daily sea surface temperatures at a shocking pace. LJ was diagnosed with heat exhaustion, with treatment and prevention being the simple regimen of moving to a cooler environment, hydration, rest, and avoiding heat exposure whenever possible. However simple, for thousands like LJ with jobs with unavoidable exposure to extreme heat, threats of heat-related illness like dehydration, heat exhaustion, and heat stroke are common and pertinent threats to their health. Despite rising daily temperatures and heat-related health outcomes, protections for these workers are actively under threat.

Continuous international temperature record-keeping began in 1880 and has been developed to the modern day where agencies collect, interpret, and collaborate on analyzing global weather patterns (1). This record-keeping has resulted in a large body of research demonstrating an undeniable trend of increasing average global temperatures since the beginning of the Industrial Revolution (2). In contrast, workplace morbidity and mortality were highest at the turn of the 20th century and improved by 90% from 1933 to 1997, during which the workforce increased more than three-fold (3). Despite this commendable progress, safety among the labor force is threatened due to rising temperatures, with heat-related illnesses accounting for roughly 37% of all weather-related fatalities over the past 30 years (4). In the face of such a threat, populations already at risk, including construction and agricultural workers, are disproportionately affected. While expanding heat-protection regulations in the workplace would be a logical implementation for the health and safety of workers, recent state legislations in Florida and Texas have banned such measures (5, 6). Preventing heat protection poses a significant threat to the morbidity and mortality of heat-vulnerable workers and other at-risk populations, and the establishment of such protections must be prioritized.

On April 11, 2024, Governor Ron DeSantis approved HB 433, “prohibiting a political subdivision from requiring employers to meet or provide heat exposure requirements beyond those required by law” (6). While it may seem like Florida would enact such a bill to standardize heat protection laws statewide, there are currently no heat protection laws in place, meaning HB 433 prevents heat protection requirements in Florida. This house bill prevents any heat protection for the 140,000+ agriculture production workers and nearly 630,000 construction workers (7, 8). As of 2018, agriculture and construction account for roughly 4.8% of all jobs in Florida; however, these values may underestimate their presence, as undocumented workers are estimated to constitute 49 percent of the farming, fishing, and forestry industries and 38 percent of the construction and extraction industry (9, 10). The state most comparable to Florida for the necessity of heat protection laws is California, which has 500,000–800,000 farmworkers, about 58 percent of whom are undocumented, and which established statewide heat protection regulations in 2005 (11–13).

An in-depth study by Gubernot et al. analyzed data from the Bureau of Labor Statistics to determine occupational heat-related fatalities in the United States between 2000 and 2010. Florida had the ninth-highest rate of 0.37 deaths per 1,000,000 workers per year, while California reported 0.24 deaths per 1,000,000 workers per year. Of note, the national average was 0.22 deaths per 1,000,000 workers per year, and of the 10 states with the highest heat-related fatality rates, zero have heat protection laws as of 2024 (14). According to the Department of Labor, agriculture and construction workers should have monetary compensation in their wages as hazard pay, given in hazardous workplace environments without sufficient protective measures (15). The insufficient inclusion of hazard pay strains workers' current standard of living and inadequately compensates for the long-term effects of chronic heat exposure. Furthermore, given the nature of most agricultural work, one can argue that the working conditions of most agricultural workers can be considered hazardous and that unclear definitions of “hazardous pay” may allow workplaces to avoid compensating workers. Instead, given the nature of their working conditions, agricultural workers in general should receive increased compensation reflected in their wages to account for the hazardous nature of their workplace setting. A study by Mix et al. performed a study on nearly 200 agriculture workers in Florida to evaluate the impact of heat on kidney function. Each worker was able to contribute up to three consecutive workdays of data, and it was found that 33% of workers had measurable acute kidney injury (AKI) on at least one of the 3 days (16). While AKI has acute dangers, repeated AKI can cause permanent damage, increasing the risk of developing chronic kidney disease, heart disease and stroke (17). While ensuring workplace safety and providing adequate compensation for hazardous environments needs to be a priority, it is also an equitable responsibility to minimize long-term health effects, especially with serious conditions like AKI that can be avoided with proper hydration. The urgency of workforce protection cannot be overstated. Florida's lack of heat protection laws puts thousands of workers at risk daily. However, if the Occupational Safety and Health Administration (OSHA) were to establish a federal requirement for heat protection, Florida workplace policies would have to comply, helping to mitigate worker risks. Under the Occupational Safety and Health Act of 1970, Federal OSHA was excluded from regulating state and local government employees (18). While this act also requires state governments to establish protections for federal employees deemed at least as effective as those set by Federal OSHA, there are currently no Federal OSHA heat-related regulations. On July 23, 2023, President Biden asked OSHA to begin issuing heat hazard alerts and discussed implementing Federal OSHA heat policies. However, President Biden also noted, “[a] workplace heat standard has long been a top priority for the Department of Labor, but rulemaking takes time,” raising the question of how long state employees will have to work without a Federal OSHA heat-related legal precedent protecting their health and safety (19).

Introduced in July 2023, ordinance file number 231454, *Creating a Heat Standard for Outdoor Workers*, aimed to implement heat-related protective regulations for Miami-Dade County, including “requiring…an approved mandatory heat exposure safety program… access to drinking water…shaded recovery periods…multilingual notice of employee rights…establishing penalties for violations of chapter…” (20). This proposed ordinance remained in discussion into 2024 but as of July 1, 2024, HB 433 barred the proposal from being established and directly resulted in its formal withdrawal. DeSantis' motivation for such a ban appears to be the prioritization of business owners retaining control over working conditions, in lieu of ensuring the safety of workers. This is supported by a comment by DeSantis after signing HB 433, in which he states, “[t]here was a lot of concern out of one county, Miami-Dade. And I don't think it was an issue in any other part of the state” (21). DeSantis reasoned allowing local heat-related policies would “impos[e] restrictions in ways that are not likely going to be feasible when you have people doing in multiple jurisdictions…it should be one rule for the state” (22, 23). The prioritization of business owners was made clear when questioned about the safety of workers, to which DeSantis replied, “businesses are able to do what they want to do” (22). Such logic contradicts the purpose of OSHA's creation, which is to establish and enforce workplace regulations that reduce health and safety risks faced by employees (24). Miami-Dade's bill was supported by workers and activists but contested by agriculture and construction employers and lobbyists. Ultimately, preemption from employers and lobbyists raised the required heat index of the bill, reducing the estimated affected days per year from 180 days down to 3 (25). While opposing regulations ensuring the safety of workers is against their wellbeing regardless of location, Miami is one of the cities in Florida, arguably the entire United States, most in need for such protective measures due to local factors. Without enforceable standards, the safety of the most vulnerable workers is at risk of being valued second to business operations, and local governments should be encouraged, not barred, to create their own standards based on the needs of their population.

Texas Governor Abbott and Florida Governor DeSantis both reasoned heat-safety regulations, if implemented at all, should be done at the state level (21). Climate records demonstrate why statewide heat regulations without allowing local ordinances interferes with the primary purpose of workplace regulations: to ensure an environment with protective measures minimizing the exposure to health risks and preventing health consequences of said risks where possible. Using the temperature California deems as necessitating heat-related policies for reference (80 degrees Fahrenheit) highlights the striking difference between Florida's largest cities, Jacksonville and Miami. Looking at NOAA weather records, in 2023, Jacksonville had 237 days where the temperature was at least 80-degrees, while Miami had 318 days (26–28). Perhaps a better indicator of heat stress is a dew point of 65-degrees Fahrenheit or greater, a level at which the NOAA describes as “becoming oppressive” (29). Based on climate, Jacksonville's lowest frequency of days with a dew point above 65-degrees is in January at 2%, while Miami's lowest is 30% in January. Jacksonville has a 100% chance of a 65-degree dew point for roughly 1 month between July and August, while Miami has ~3 months (30). Evaluating the percentage of time at “miserable” comfort levels, meaning a dew point >75-degrees Fahrenheit, demonstrates a maximum frequency of roughly 15% in Jacksonville and just over 40% in Miami (31). Such unique environmental factors impacting the heat index experienced in Miami demonstrate the necessity of both universal heat protection laws and the ability for local governments to establish higher protection standards based on their local climate (30). Evaluating the percentage of time at “miserable” comfort levels, meaning a dew point >75 degrees Fahrenheit, demonstrates a maximum frequency of roughly 15% in Jacksonville and just over 40% in Miami (31). Such unique environmental factors impacting the heat index experienced in Miami demonstrate the necessity of universal heat protection laws and the ability for local governments to establish higher protection standards. These records demonstrate why statewide heat regulations without the possibility of local ordinances interfere with the primary purpose of workplace regulations: to ensure a workplace environment with protective measures against health risks and removal of said risks where possible.

## Discussion

As average global surface temperatures continue to rise, there remains an urgent need to protect the health of populations most susceptible to the consequences of extreme heat exposure, especially in our workforce. The potential impact of securing heat protection laws is immense, as they can significantly reduce the risk of heat-related illnesses and fatalities, ensuring a safer working environment for thousands of workers exposed to hazardous heat. In our hospitals and healthcare centers, providers should be aware of the interplay between climate, the built environment, and acute and chronic health in our hospitals and healthcare centers. This intersection of factors allows solutions to be pursued from multiple disciplines. Legislation and projects that aim to improve city livability and reduce heat stress, such as increasing the number of shaded areas, green spaces, and public water sources, can be encouraged by contacting local and state representatives. While these improvements would have the greatest immediate effect on city inhabitants, reducing the urban island heat effect would directly lower heat stress for city construction workers. For agriculture workers, anyone can take action by learning about locally supplied produce and choose those that integrate worker safety and compensation a priority, such As those in the fair food program or certified by independent reviewers like ethical consumer. Our legislative bodies, healthcare providers, and public health professionals play a vital role in highlighting heat as an important determinant of health, and any individual can advocate for laws and regulations that make heat protection a default in our workspaces rather than an afterthought.

## References

[1] Why does the temperature record shown on your “Vital Signs” page begin at 1880? - NASA Science. NASA. Available at: https://science.nasa.gov/climate-change/faq/why-does-the-temperature-record-shown-on-your-vital-signs-page-begin-at-1880/ (accessed June 17, 2024).

[2] Lindsey R Dahlman L. Climate Change: Global Temperature. (2024). Available at: https://www.climate.gov/news-features/understanding-climate/climate-change-global-temperature# (accessed June 17, 2024).

[3] Centers for Disease Control and Prevention. Achievements in public health, 1900-1999: improvements in workplace safety – United States, 1900-1999. MMWR Morb Mortal Wkly Rep. (1999) 48:461–9. 10428100

[4] National Oceanic and Atmospheric Administration. Weather Related Fatality and Injury Statistics. NOAA. Available at: https://www.weather.gov/hazstat/ (accessed June 17, 2024).

[5] Burrows. H.B. No 2127. Texas State Legislature (2023). Available at: https://capitol.texas.gov/tlodocs/88R/billtext/pdf/HB02127F.pdf#navpanes=0 (accessed June 14, 2024).

[6] CS/CS/HB 433: Employment Regulations Florida Florida House of Representatives. (2024). Available at: https://flsenate.gov/Session/Bill/2024/433/?Tab=BillHistory

[7] Florida's Agriculture and Food System Fast Facts. University of Florida - Institute of Food and Agricultural Sciences. (2022). Available at: https://branding.ifas.ufl.edu/downloads/uploads/Extension%20Brochures/IFAS/Florida-Agriculture-Food-System-Fast-Facts.pdf (accessed July 3, 2024).

[8] Federal Reserve Bank of St. Louis. All Employees: Construction in Florida. US Bureau of Labor Statistics. Available at: https://fred.stlouisfed.org/series/SMU12000002000000001A (accessed June 14, 2024).

[9] Division of Occupational Employment and Wage Statistics. Occupational Employment and Wage Statistics. (2024). Available at: https://www.bls.gov/oes/2023/may/oes_fl.htm (accessed June 13, 2024).

[10] American Immigration Council. Immigrants in Florida. (2020). Available at: https://www.americanimmigrationcouncil.org/sites/default/files/research/immigrants_in_florida.pdf (accessed June 14, 2024).

[11] California Department of Food and Agriculture. California Agriculture Statistics Review. State of California. Available at: https://www.cdfa.ca.gov/statistics/pdfs/resourcedirectory_2011-2012.pdf (accessed June 20, 2024).

[12] Cha P. Health Care Access among California's Farmworkers. (2022). Available at: https://www.ppic.org/publication/health-care-access-among-californias-farmworkers/ (accessed June 19, 2024).

[13] California Department of Industrial Relations (DIR). T8CCR 3395. Heat Illness Prevention in Outdoor Places of Employment (2005). Available at: https://www.dir.ca.gov/title8/3395.html (accessed June 12, 2024).

[14] Gubernot DM Anderson GB Hunting KL. Characterizing occupational heat-related mortality in the United States, 2000–2010: an analysis using the census of fatal occupational injuries database. Am J Ind Med. (2015) 58:203–11. 10.1002/ajim.22381 25603942 PMC4657558

[15] Hazard Pay. Wage and Hour Division. Available at: https://www.dol.gov/general/topic/wages/hazardpay (accessed June 19, 2024).

[16] Mix J Elon L Vi Thien Mac V Flocks J Economos E Tovar-Aguilar AJ. Hydration status, kidney function, and kidney injury in florida agricultural workers. J Occup Environ Med. (2018) 60:e253–60. 10.1097/JOM.0000000000001261 29271837

[17] National Kidney Foundation Patient Education Team (NPE). Acute Kidney Injury (AKI). National Kidney Foundation. (2024). Available at: https://www.kidney.org/kidney-topics/acute-kidney-injury-aki (accessed October 4, 2024).

[18] Fairfax RE. Federal OSHA has no jurisdiction over State, municipal, or volunteer fire departments. Occupational Safety and Health Administration: Standard Interpretations. Washington, DC: US Department of Labor (2006).

[19] Department Department of Labor announces hazard alert steps up enforcement as extreme heat endangers workers across the nation. U.S. Department of Labor (2023). Available at: https://www.osha.gov/news/newsreleases/national/07272023 (accessed July 3, 2024).

[20] Ordinance 231454: Creating a Heat Standard for Outdoor Workers, Miami-Dade County Legislature. (2023). Available at: https://www.miamidade.gov/govaction/matter.asatter=231454&file=true&fileAnalysis=false&yearFolder=Y2023 (accessed July 3, 2024).

[21] B A. Florida blocks heat protections for workers right before summer. NPR. Available at: https://www.npr.org/2024/04/12/1244316874/florida-blocks-heat-protections-for-workers-right-before-summer (accessed October 2, 2024).

[22] Gancarski AG. Ron DeSantis says business couldn't handle 'patchwork' of local heat protections for workers. Extensive Enterprises Media. Available at: https://floridapolitics.com/archives/686189-ron-desantis-patchwork-heat/ (accessed October 2, 2024).

[23] Saunders J. Hot topic: Florida barred heat protection for workers, now Feds are stepping in. Orlando Sentinel. Available at: https://www.orlandosentinel.com/2024/07/02/hot-topic-florida-barred-heat-protection-for-workers-now-feds-are-stepping-in/ (accessed October 2, 2024).

[24] Occupational Safety and Health Administration [OSHA]. OSHA's 30th Anniversary. US Department of Labor. Available at: https://www.osha.gov/aboutosha/30-years#:~:text=OSHA%20was%20created%20because%20of,and%20deaths%20in%20the%20workplace (accessed October 3, 2024)..

[25] Miznazi A. Miami-Dade's ends push to protect outdoor workers from Florida heat. WLRN. (2024). Available at: https://health.wusf.usf.edu/health-news-florida/2024-03-22/miami-dades-ends-push-to-protect-outdoor-workers-from-florida-heat (accessed July 6, 2024).

[26] Extreme Weather Watch. Number of Days of 80 Degrees Fahrenheit in Miami by Year. H Brothers Inc. Available at: https://www.extremeweatherwatch.com/cities/miami/yearly-days-of-80-degrees (accessed July 11, 2024).

[27] Extreme Weather Watch. Number of Days of 80 Degrees Fahrenheit in Jacksonville by Year. H Brothers Inc. Available at: https://www.extremeweatherwatch.com/cities/jacksonville/yearly-days-of-80-degrees (accessed July 11, 2024).

[28] National Weather Service. NOAA Online Weather Data (2024). Available at: https://www.weather.gov/wrh/climate?wfo=mfl (accessed July 12, 2024).

[29] NOaAA. Dew Point vs Humidity. US Department of Commerce. Available at: https://www.weather.gov/arx/why_dewpoint_vs_humidity#:~:text=less%20than%20or%20equal%20to,in%20the%20air%2C%20becoming%20oppressive (accessed October 3, 2024).

[30] Compare the Climate and Weather in Miami and Jacksonville - Weather Spark. Weather Spark. Available at: https://weatherspark.com/compare/y/18622~17779/Comparison-of-the-Average-Weather-in-Miami-and-Jacksonville (accessed July 8, 2024).

[31] Climate and Average Weather Year Round in Miami. Weather Spark. Available at: https://weatherspark.com/y/18622/Average-Weather-in-Miami-Florida-United-States-Year-Round#Sections-Humidity (accessed July 8, 2024).

